# Supplementary material for: Integrating Single-Cell RNA-Seq and Bulk RNA-Seq Data to Explore the Key Role of Fatty Acid Metabolism in Breast Cancer
Source: Int J Mol Sci. 2023 Aug 25;24(17):13209. doi: 10.3390/ijms241713209 (PMC10487665; doi:10.3390/ijms241713209)
Supplement: Supplementary file 1 [file ijms-24-13209-s001.zip › Supplementary Figure.pdf]

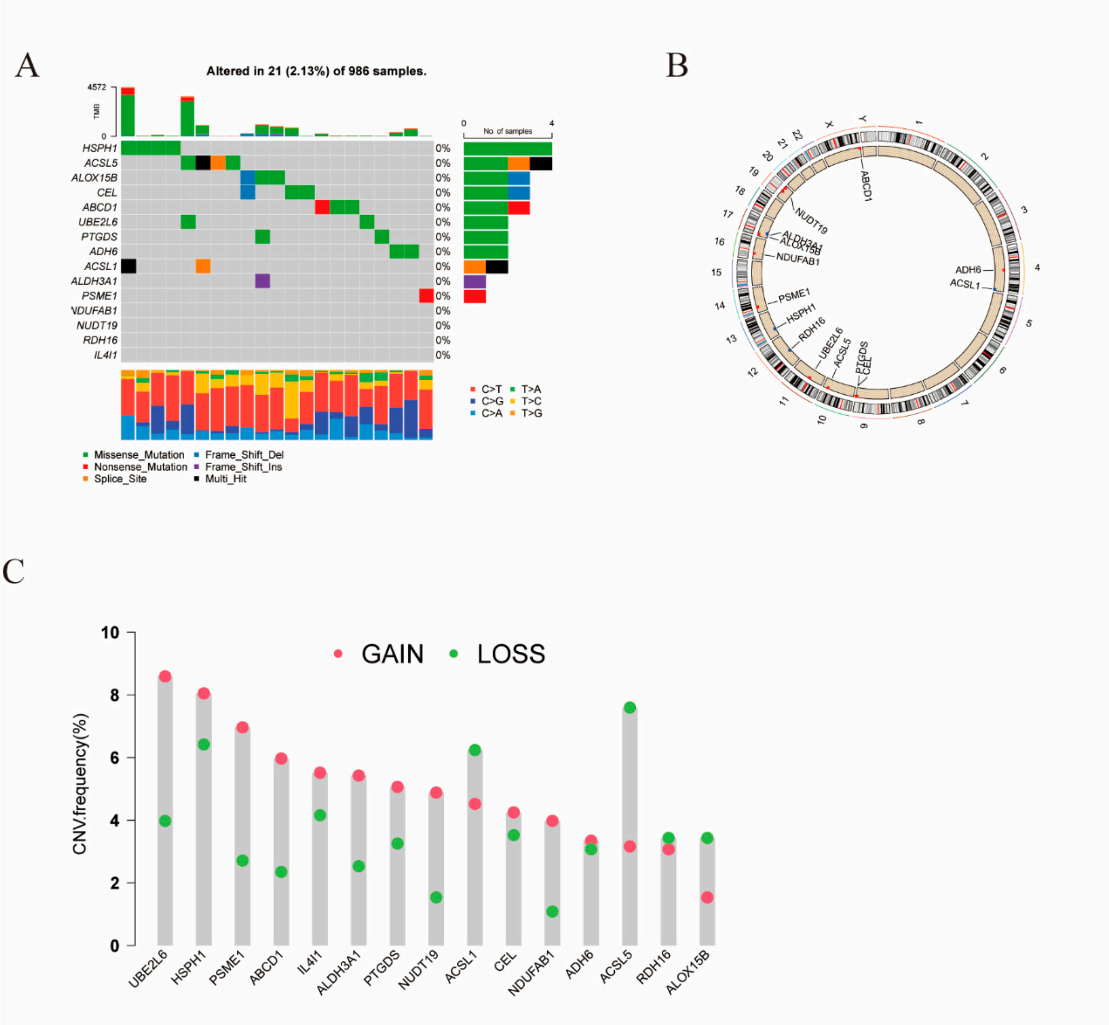

Supplementary Figure S1| Genetic variation and expression of 15 FMGs in BRCA. (A) Genetic alteration of FMGs query. Only 21 out of 986 samples were mutated. (B)The location of CNV alterations in FMGs represents CNV amplification and green dots represent CNV deletion. (C)Compared with other FAM-related genes, ACSL1, ACSL5 and ALOX15B have a higher frequency of CNV deletion, while UBE2L6, HSPH1 and PSME1 have a higher frequency of CNV amplification.

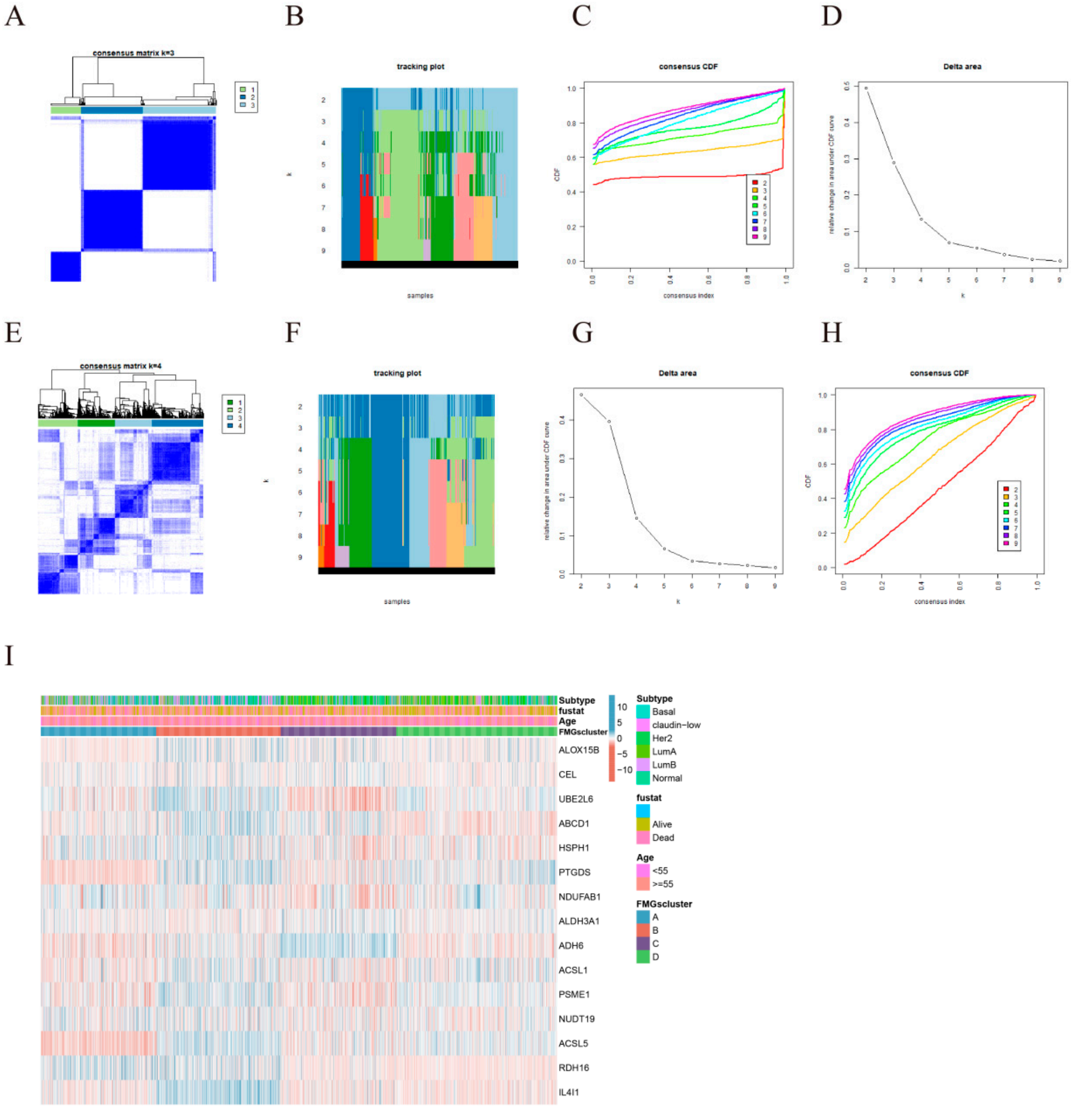

Supplementary Figure S2 |Unsupervised clustering identified three distinct FMGs clusters and validated the clustering effectiveness through the METABRIC dataset. (A) Consensus clustering matrix for k = 3. (B) Distribution of each sample across different clusters as k ranges from 2 to 9. (C) Cumulative Distribution Function (CDF) of consensus clustering for k = 2–10. (D) Relative change in the area under the CDF curve for k = 2–9. (E) Consensus clustering matrix for k = 4 in the METABRIC dataset. (F) Distribution of each sample across different clusters in the METABRIC dataset as k ranges from 2 to 9. (G) CDF of consensus clustering for k = 2–10 in the METABRIC dataset. (H) Relative change in the area under the CDF curve for k = 2–9 in the METABRIC dataset. (I) Heatmap displays the correlation between clustering and clinical information in the METABRIC dataset.

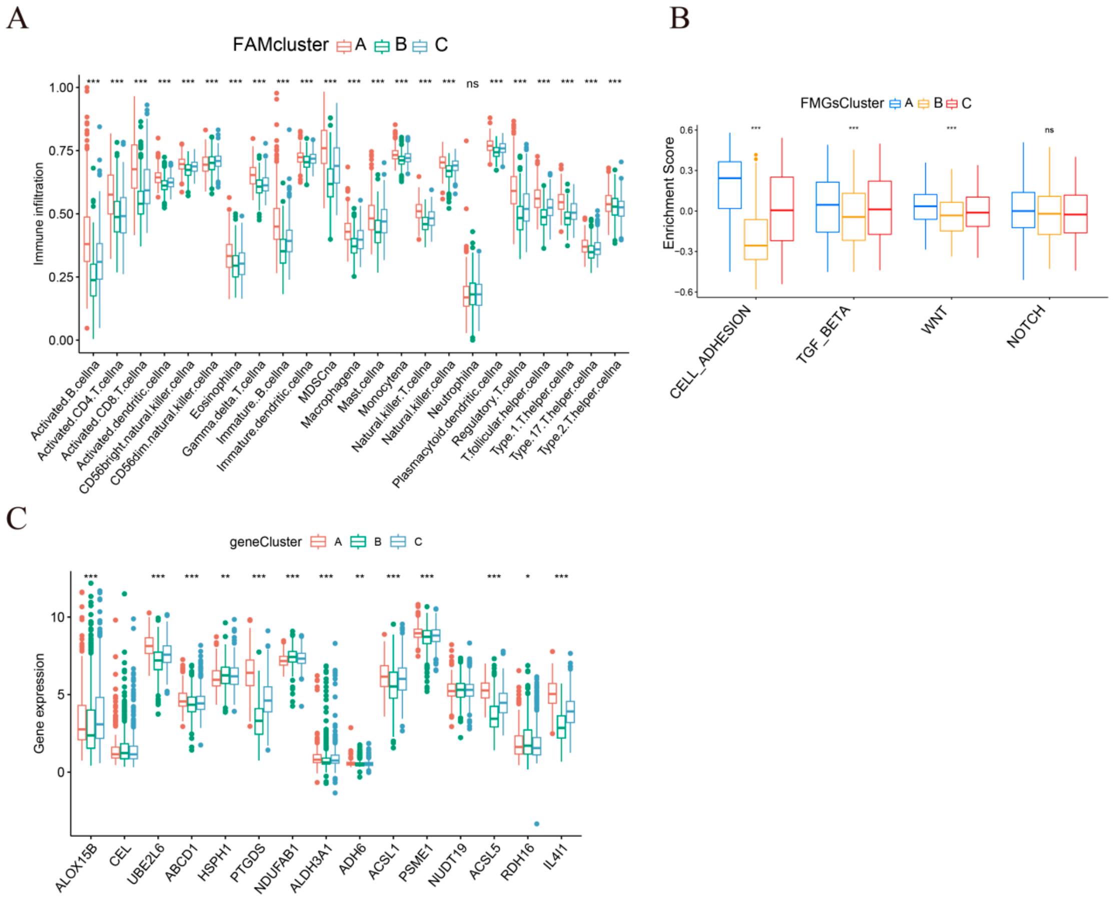

Supplementary Figure S3 |(A) The abundance of each TME infiltrating cell in the three FMGs-Cluster cells. (B) Three distinct FMGs modification patterns exhibit differences in stroma-activated pathways, including CELL_ADHESION, TGF beta, WNT, and NOTCH. The asterisks represented the statistical p-value (*P < 0.05; **P < 0.01; ***P < 0.001).(C) Boxplot demonstrating differences in 15 fatty acid metabolism genes between the three gengcluster groups. (*P < 0.05; **P < 0.01; ***P < 0.001).

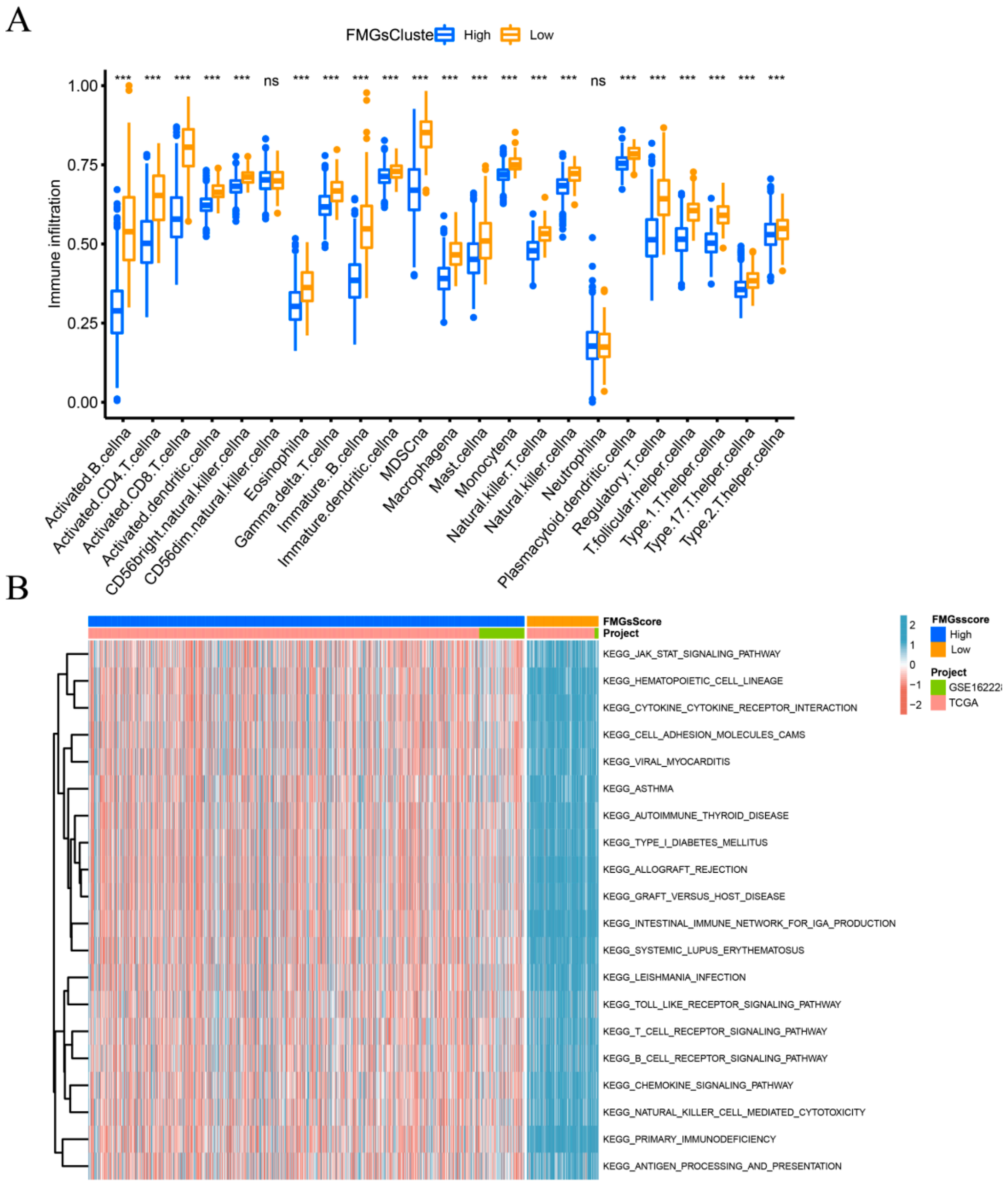

Supplementary Figure S4|(A) Differences in immune infiltrating cells in the high and low subgroups of FMGsScore. (B) GSVA enrichment analysis between high and low FMGsScore subgroups (*P < 0.05, **P < 0.01, ***P < 0.001. ns means the difference was not statistically significant).

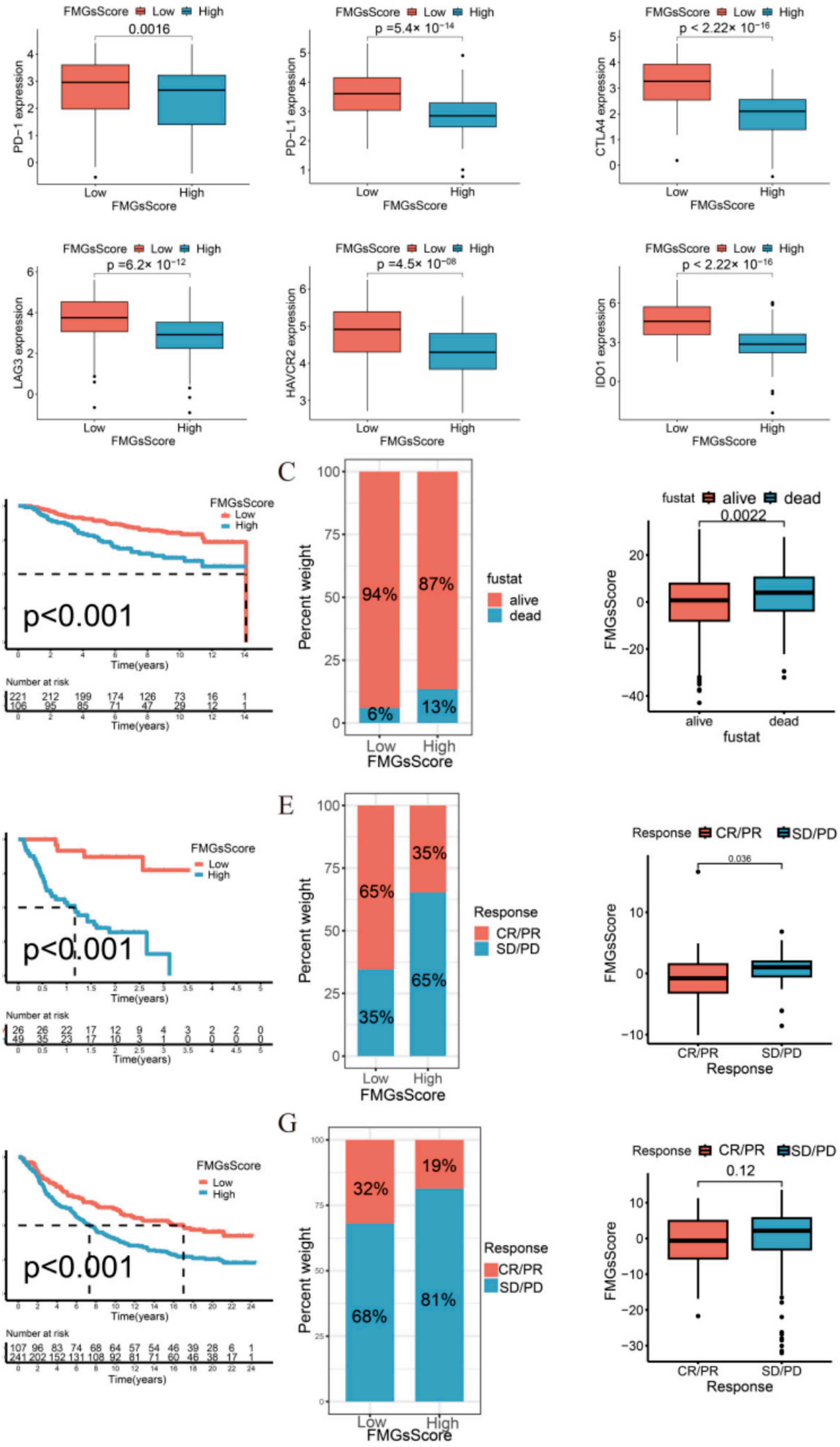

FMGsScore Low High
0.0016
PD−1 expression
p =5.4× 10−14
PD−L1 expression
p < 2.22× 10−16
CTLA4 expression
p =6.2× 10−12
LAG3 expression
p =4.5× 10−08
HAVCR2 expression
p < 2.22× 10−16
IDO1 expression
FMGsScore
C
p<0.001
Time(years)
Number at risk
221 212 199 174 126 73 16 1
106 95 85 71 47 29 12 1
Percent weight
94%
87%
6%
13%
fustat
alive
dead
fustat alive dead
0.0022
FMGsScore
E
p<0.001
26 26 22 17 12 9 4 3 2 2 0
49 35 23 17 10 3 1 0 0 0 0
65%
35%
Response
CR/PR
SD/PD
Response CR/PR SD/PD
0.036
G
107 96 83 74 68 64 57 54 46 39 28 6 1
241 202 152 131 108 92 81 71 60 46 38 17 1
32%
68%
19%
81%
0.12

Supplementary Figure S5 |Validation of the accuracy of FMGsScore. (A) Differences in expression of six immune checkpoint genes in the FMGsScore high and FMGsScore low groups in the GSE20685 dataset. (B) Kaplan-Meier curves of OS in the FMGsScore high and FMGsScore low groups in the GSE20685 dataset. (C) Proportion of patients with survival status in the high FMGsScore group and low FMGsScore group in the GSE20685 dataset and box plots. (D) Survival analysis of patients with high and low FMGsScore in the melanoma cohort. (E) Proportion of patients who responded or did not respond to PD-L1 blockade therapy in the FMGsScore high and FMGsScore low groups in the melanoma cohort and box plots. (F) Kaplan-Meier curves showing OS in the high FMGsScore and low FMGsScore groups in the IMvigor210 cohort. (G) Proportion of patients responding to immune checkpoint blockade treatment in the high FMGsScore and low FMGsScore groups in the IMvigor210 cohort and box plots.

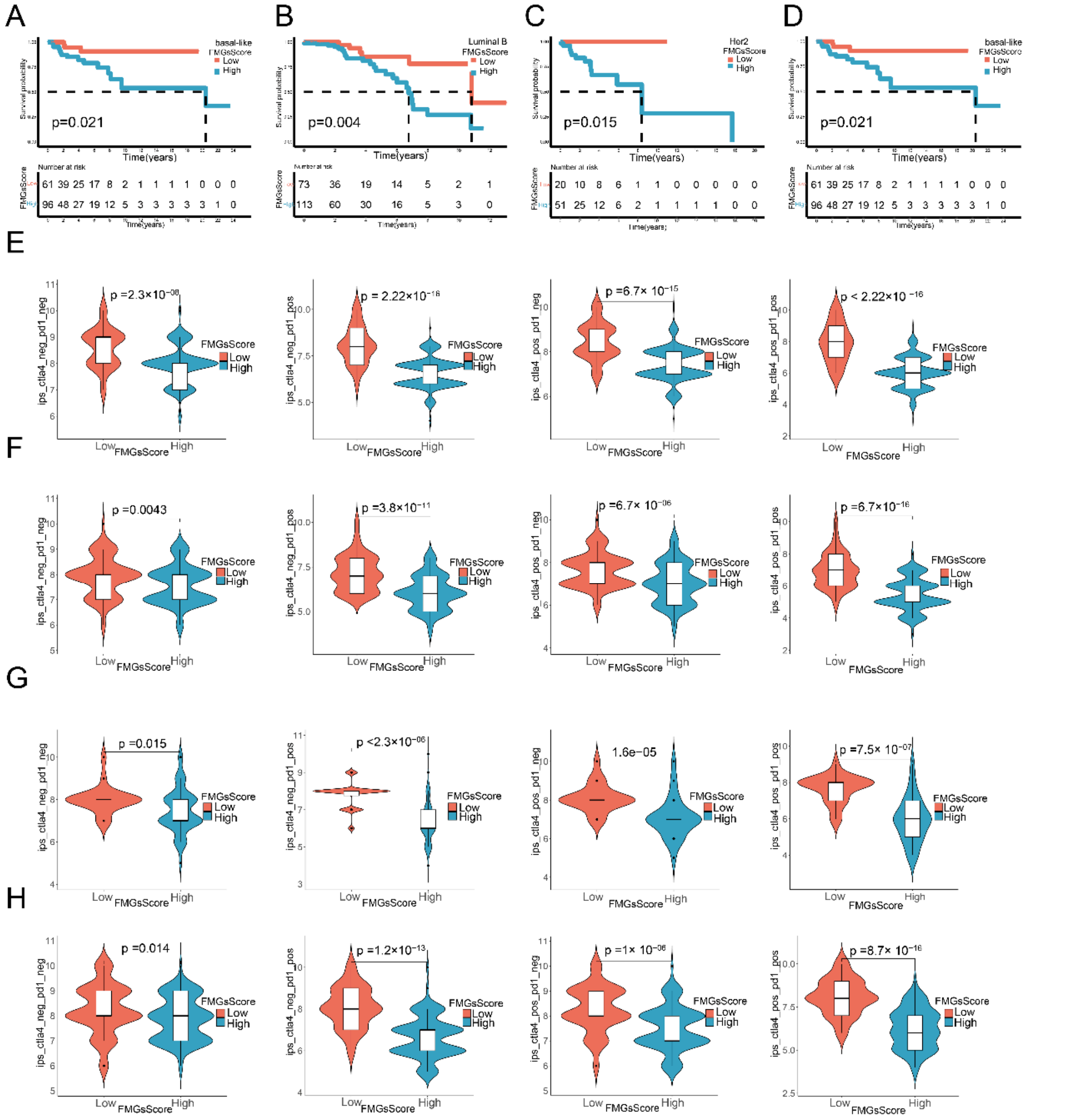

Supplementary Figure S6 |Validating the impact of FMGsScore across different subtypes of breast cancer. (A-D) K-M survival curves were generated to compare the prognosis between groups with high and low FMGsScore scores in different breast cancer subtypes. (E) In the Luminal A subtype FMGsScore was utilized to predict the immunotherapy response. (F) In the Luminal B subtype. FMGsScore was used for predicting the immunotherapy response. (G), In the Basal-like subtype, FMGsScore was employed to predict the immunotherapy response. (H)In the HER2-enriched subtype, FMGsScore was utilized for predicting the immunotherapy response.
