# Supplementary material for: Integrating Single-Cell RNA-Seq and Bulk RNA-Seq Data to Explore the Key Role of Fatty Acid Metabolism in Breast Cancer
Source: Int J Mol Sci. 2023 Aug 25;24(17):13209. doi: 10.3390/ijms241713209 (PMC10487665; doi:10.3390/ijms241713209)
Supplement: Supplementary file 1 [file ijms-24-13209-s001.zip › Table S1.pdf]

Table S1. Oligonucleotides used in research

Oligonucleotides	Nucleotide sequence (5'-3')
**siRNA**	
SiRNA-NC	UUCUCCGAACGUGUCACGUTT
Si-NDUFAB1	GACCUGGGCUUAGACAGUUTT
**Primer**	
β -acting	CCTGGCACCCAGCACAAT (forward)
	GGGCCGGACTCGTCATACT (reverse)
NDUFAB1	ATGGCGTCTCGTGTCCTTTC(forward)
	AACCTGCGCGAGCACTAAG (reverse)
